# Advances in Molecular Genetics and the Molecular Biology of Deafness

**DOI:** 10.1155/2016/5629093

**Published:** 2016-07-20

**Authors:** Shin-ya Nishio, Isabelle Schrauwen, Hideaki Moteki, Hela Azaiez

**Affiliations:** ^1^Shinshu University School of Medicine, Matsumoto, Nagano 390-8621, Japan; ^2^Translational Genomics Research Institute, Phoenix, AZ 85004, USA; ^3^Department of Otolaryngology, University of Iowa, Iowa City, IA 52246, USA

Congenital sensorineural hearing loss is the most common sensory disorder, with approximately 1 in every 1000 newborns in developed countries suffering from severe-to-profound hearing loss. At least half of those cases are attributable to genetic causes with more than 90 causative genes identified to date reflecting the complex clinical and genetic landscapes of hereditary hearing loss [1, 2].

Recent advances in molecular genetics technologies, notably next-generation sequencing (NGS), have drastically accelerated the identification of novel genes involved in hearing mechanism and expanded the mutational spectrum of known deafness-causing genes [3–9]. In addition to NGS, recent progress in genome editing, embryonic stem cells, and induced pluripotent stem cells has opened a new gate to a fast and thorough characterization and understanding of the precise functions and mechanisms involved in the biology of hearing and deafness.

This special issue is to exhibit the advances and recent progress in the fields of molecular genetics and molecular biology of hearing and deafness.

The review paper by S. Kitajiri and T. Katsuno (Kyoto University) summarized the importance of the tricellular tight junction proteins (tricellulin, occludin, ILDR1, and the angulin family) in the inner ear by acting as a barrier separating the endolymphatic and perilymphatic spaces, which is essential for the generation and maintenance of the endocochlear potential.

M. Hosoya et al. described the cochlea distribution patterns of KIAA1199 proteins in a nonhuman primate, the common marmoset (*Callithrix jacchus*).* KIAA1199* has been reported as a cause of progressive hearing loss, but its spatial expression showed different and distinct patterns in mouse and rat cochlea. In this report, M. Hosoya at al. showed a more widespread KIAA1199 protein expression in the marmoset. These results are of importance for further investigation and elucidation of the functional role of KIAA1199 in primate cochlea.

Developing novel diagnostic tools that are tailored to specific ethnicities is crucial for a cost-effective genetic screening for deafness. In this issue, F. Zhang et al. described their multiplex genetic screening system “SNPscan assay” used to screen a total of 115 known mutations in* GJB2*,* SLC26A4,* and mtDNA 12SrRNA.

Cochlear implantation (CI) is the most important and effective treatment for patients with profound sensorineural hearing loss. However, outcomes vary among patients due to several reasons, one being the heterogeneous nature of the clinical as well as genetic etiology of hearing loss. H. Koyama et al. reported the CI outcomes in five patients with Waardenburg syndrome. They showed that CI is a good and suitable treatment option for Waardenburg syndrome cases.

Statins are inhibitors of the 3-hydroxy-3-methylglutaryl-coenzyme A reductase and widely used as cholesterol-lowering drugs. However, in the last decade, conflicting data about the effect of statins on neuronal cells and the auditory system has been published. K. Leitmeyer et al. studied the effect of simvastatin on spiral ganglion neurons explants in vitro and showed its neurotoxic effect that seems to be at least partially mediated by the mevalonate pathway.

In this special issue, we collected both basic and clinical original research articles stimulating the continuing efforts to understand the mechanisms of deafness and hearing systems. It is our wish to increase interest in this field and further accelerate future treatment options based on solid basic research.
